# BRASH (Bradycardia, Renal Failure, Atrioventricular Blockage, Shock, and Hyperkalemia) Syndrome: Diagnostic and Therapeutic Challenges in a Rare Clinical Entity

**DOI:** 10.7759/cureus.94332

**Published:** 2025-10-11

**Authors:** Abdulmohsen Aljishi, Mahmoud Saad

**Affiliations:** 1 Critical Care Medicine, Qatif Central Hospital, Qatif, SAU

**Keywords:** atrioventricular nodal blockade, bradycardia, brash syndrome, chronic kidney disease, heart failure, hyperkalemia

## Abstract

Bradycardia, renal failure, atrioventricular nodal blockade, shock, and hyperkalemia form a rare but potentially life-threatening constellation known as BRASH syndrome. It typically occurs in patients with chronic kidney disease and is often precipitated by the use of AV nodal blocking agents.

We present the case of a 68-year-old male with advanced chronic kidney disease (CKD stage 5), heart failure with preserved ejection fraction (HFpEF), emphysema, and hypothyroidism who developed severe bradycardia (heart rate: 28 beats per minute) and hypotension (blood pressure: 88/54 mmHg). Laboratory evaluation revealed profound hyperkalemia (7.4 mmol/L), acute kidney injury with a creatinine level of 1,538 μmol/L (baseline 494 μmol/L), and severe metabolic acidosis (pH 6.86). The electrocardiogram demonstrated an idioventricular rhythm with a heart rate of 30 beats per minute and absent P waves. Despite initial refractory instability, the patient was stabilized with prompt electrolyte correction, inotropic support, discontinuation of AV nodal blockers, and urgent hemodialysis. His clinical condition improved significantly, with normalization of potassium, resolution of bradycardia, and recovery of renal function.

This case highlights the diagnostic complexity of BRASH syndrome and underscores the therapeutic importance of early recognition and comprehensive management. Clinicians should maintain a high index of suspicion in patients with renal dysfunction receiving AV-nodal blockers who present with severe bradycardia out of proportion to serum potassium levels, as timely intervention may be lifesaving.

## Introduction

Unexplained bradycardia in the setting of acute kidney injury and hypotension often presents a diagnostic dilemma. Clinicians may initially suspect sepsis, drug toxicity, or isolated hyperkalemia, yet standard management for these conditions is not always effective. The interplay of renal dysfunction, electrolyte imbalance, and nodal-blocking medications can create a self-perpetuating cycle that amplifies cardiovascular instability [1].

Recognition of this cycle as BRASH (Bradycardia, Renal Failure, Atrioventricular (AV) Blockage, Shock, and Hyperkalemia) syndrome has provided a unifying framework to explain cases that previously defied clear categorization. Although still rarely reported, it is increasingly recognized in patients with chronic kidney disease, who are particularly vulnerable due to impaired potassium clearance, polypharmacy, and underlying cardiovascular disease [2-4].

Our case of a 68-year-old man with chronic kidney disease (CKD) who developed BRASH syndrome while on carvedilol and amlodipine illustrates the importance of early recognition and targeted therapy. Prompt pharmacological and dialytic interventions restored hemodynamic stability and renal function, underscoring the need for clinical awareness in high-risk patients.

## Case presentation

A 68-year-old male with advanced chronic kidney disease (CKD stage 5) and a baseline creatinine of 494 μmol/L (estimated glomerular filtration rate (eGF) < 15 mL/min/1.73 m²), heart failure with preserved ejection fraction (HFpEF, 50%) confirmed on echocardiography six months prior, emphysema, and hypothyroidism presented with worsening fatigue, anorexia, oliguria, and dyspnea for five days. His ongoing medications included carvedilol, amlodipine, sodium bicarbonate, calcium carbonate, furosemide, L-thyroxine, rosuvastatin, darbepoetin, and activated vitamin D (alfacalcidol). The patient had been taking all medications at stable doses for more than one year, with no recent introductions or up-titration. His family brought him to the hospital due to progressive worsening of symptoms. Upon arrival at the emergency department, he was in hemodynamic instability, with a blood pressure of 88/54 mmHg, heart rate of 28 beats per minute, respiratory rate of 28 breaths per minute, and oxygen saturation of 90% on room air. Physical examination revealed bilateral lower-extremity edema, and pulmonary crackles were noted on auscultation. Neurologically, he was alert and oriented, with a Glasgow Coma Scale (GCS) score of 15.

**Figure 1:**
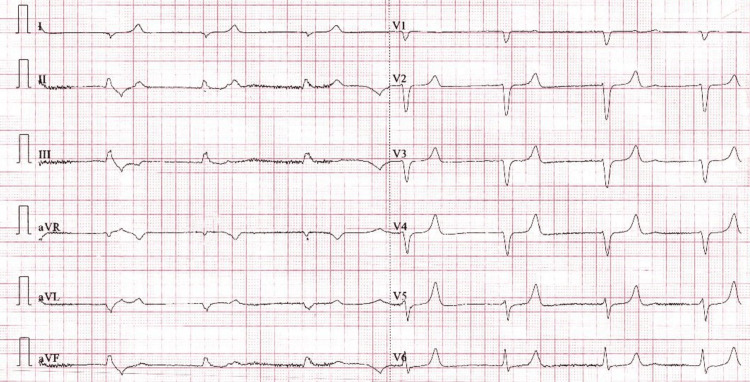
Admission ECG showing complete heart block.

Laboratory tests revealed significant electrolyte imbalances, with potassium at 7.6 mmol/L and creatinine at 1,538 μmol/L (from a baseline of 494 μmol/L). Thyroid function tests showed an elevated thyroid-stimulating hormone (TSH) level of 132 μIU/mL, and severe metabolic acidosis with a pH of 6.86 (Table 1). The initial electrocardiogram (ECG) demonstrated an idioventricular rhythm with a heart rate of 30 beats per minute and absent P waves (Figure 1). Echocardiography revealed borderline left ventricular dilation, an ejection fraction of 50%, mild global hypokinesia, and grade II diastolic dysfunction.

**Table 1:** Laboratory values from baseline, admission, and discharge. TSH: thyroid-stimulating hormone; Hb: hemoglobin; HCO3: bicarbonate; pCO2: partial pressure of carbon dioxide

Parameter	Baseline	Admission	Discharge	Reference value
Creatinine (μmol/L)	494	1538	469	53-106
Potassium (mmol/L)	5.6	7.6	4.1	3.5-5.3
Sodium (mmol/L)	139	135	138	135-153
WBC (*9/L)	7.45	5.64	5.57	4-10
Hb (mg/dL)	9.7	10.7	9.38	13-17
TSH (μIU/L)	132	150	150	0.55-4.78
Random blood sugar (mmol/L)	5.3	11.5	4.8	3.33-9.99
Blood gas pH	-	6.86	7.42	7.36-7.41
HCO3 (mmol/L)	-	6.2	23.7	22-26
pCO2 (mmHg)	-	31.2	36.6	35-40
Lactate (mmol/L)	-	0.5	0.5	Less than 1

Hyperkalemia management included aggressive calcium replacement, insulin-dextrose therapy, nebulized salbutamol, and sodium bicarbonate to correct metabolic acidosis and promote potassium clearance. Inotropic agents, including dopamine, norepinephrine, and epinephrine, were administered to stabilize heart rate and maintain adequate organ perfusion. Emergency hemodialysis was initiated, and culprit medications (carvedilol and amlodipine) were discontinued to prevent further atrioventricular nodal blockade. Empiric stress-dose hydrocortisone was started alongside thyroid hormone replacement due to initial concern for possible myxedema coma. However, as the patient remained alert, normothermic, and hemodynamically responsive after correction of hyperkalemia, myxedema coma was ruled out, and hydrocortisone was discontinued. Although septic shock was initially suspected, it was excluded based on the absence of inflammatory markers and negative culture results, and therefore, antibiotics were not administered. Over the following six days, the patient’s potassium levels normalized, bradycardia resolved, and hemodynamic stability improved. At discharge, potassium was 4.1 mmol/L, and creatinine had decreased to 469 μmol/L. His revised medication regimen included furosemide, calcium carbonate, and thyroxine, with a plan for close nephrology and cardiology follow-up.

## Discussion

BRASH syndrome is a rare clinical pentad characterized by bradycardia, renal failure, atrioventricular (AV) nodal blockade, shock, and hyperkalemia. It has emerged as a significant clinical entity since its initial description in 2016 by Farkas et al. [1]. A systematic review of 70 published cases reported a mean patient age of 69 years, with beta-blockers implicated in 75% of cases and an in-hospital mortality rate of 5.7%, underscoring the critical need for early recognition and timely intervention to improve outcomes [4]. This syndrome is particularly prevalent among patients with multiple comorbidities, especially hypertension (71%), diabetes mellitus (48%), and chronic kidney disease (44%) [4]. Similar associations have also been noted in patients with pre-existing cardiac disease, including heart failure and coronary artery disease, in earlier reports [1-3].

**Figure 2:**
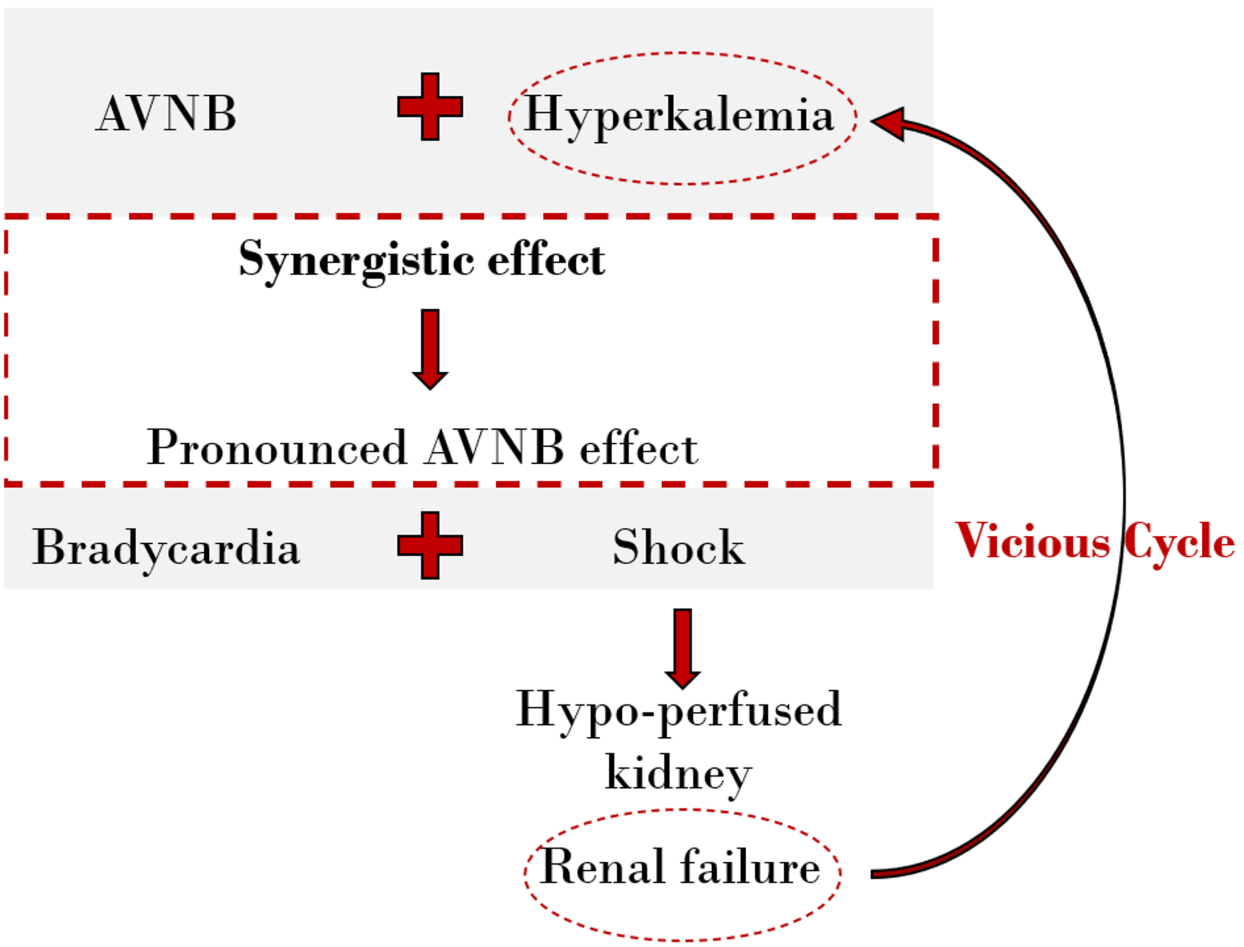
Illustration of the pathophysiological vicious cycle of BRASH syndrome. Hyperkalemia and atrioventricular (AV)-nodal blockade (AVNB) synergistically cause bradycardia, reduced cardiac output, and eventually shock. The ensuing fall in renal perfusion aggravates kidney injury, leading to further potassium retention and drug accumulation, thereby perpetuating the cycle. Redrawn and modified from the concept presented in Arif et al. [2]. BRASH: Bradycardia, Renal Failure, Atrioventricular Blockage, Shock, and Hyperkalemia

BRASH syndrome develops from a synergistic interaction between hyperkalemia, AV nodal blockade, and renal failure. Hyperkalemia and AV-nodal blockade amplify each other’s effects, resulting in profound bradycardia and hypotension. This establishes a self-perpetuating cycle in which reduced cardiac output impairs renal perfusion, further exacerbating hyperkalemia and hemodynamic instability (Figure 2) [1-3].

Understanding the pathophysiology, clinical presentation, and management of BRASH syndrome is essential, particularly in differentiating it from other critical conditions such as sepsis and myxedema coma. Although these disorders may share overlapping clinical features, their underlying mechanisms and treatment strategies are fundamentally distinct (Table 2) [3-9].

**Table 2:** Differential diagnoses considered in the evaluation of BRASH syndrome. BRASH (Bradycardia, Renal Failure, Atrioventricular (AV) Blockage, Shock, and Hyperkalemia); AV: atrioventricular

Condition	Key Features	How It Differs from BRASH Syndrome
Sepsis [3-6]	Fever, leukocytosis, hypotension, multiorgan dysfunction	Driven by infection; not primarily related to hyperkalemia or AV nodal blockade.
Myxedema Coma [3,10]	Bradycardia, hypothermia, altered mental status, pericardial effusion	Caused by severe hypothyroidism; lacks hyperkalemia and AV nodal blocker involvement.
Pure Hyperkalemia [1,3,7]	Peaked T waves, wide QRS, flaccid paralysis	Hyperkalemia is present, but without the synergy of AV nodal blockers and renal failure.

Diagnosis of BRASH syndrome primarily relies on clinical manifestations, electrocardiogram findings, and metabolic assessments while excluding other critical conditions [8]. Distinguishing BRASH from pure hyperkalemia is key, as patients with BRASH may present with only mild or moderate hyperkalemia yet develop severe bradycardia without the classic ECG changes of hyperkalemia [1]. Sepsis and myxedema coma may mimic aspects of BRASH but differ in etiology, pathophysiology, and treatment approaches [1,3-5,7,10] (Table 2).

Management of BRASH syndrome requires a comprehensive approach targeting the entire pathophysiologic cycle. The therapeutic cornerstone is the prompt correction of hyperkalemia and stabilization of cardiac conduction with intravenous calcium, followed by insulin-dextrose, β₂-agonists, and renal replacement therapy when indicated [1,3,6,8].

Fluid resuscitation should be judicious, as most patients are volume-depleted yet remain at risk of fluid overload due to underlying renal dysfunction [5]. In persistent hypotension, catecholamines such as epinephrine or isoproterenol are recommended for their combined chronotropic and inotropic effects, effectively reversing AV-nodal blockade [1-6]. Adrenergic agents were utilized in approximately 65% of reported cases, with pacing required only for refractory bradycardia [4].

Collectively, the evidence supports early correction of hyperkalemia, cautious fluid therapy, and timely catecholamine use, while reserving temporary pacing for non-responders [1,8]. Pacing is infrequently required in BRASH syndrome; approximately 33% of patients undergo temporary pacing, and none require permanent pacemaker implantation [4].

Pacing and routine Advanced Cardiac Life Support (ACLS) interventions are often ineffective in BRASH syndrome because the underlying mechanism is metabolic-pharmacologic rather than purely electrical. Hyperkalemia and AV-nodal blockade render the myocardium unresponsive to pacing stimuli until corrected. Metabolic stabilization, together with catecholamine therapy, restores electrical responsiveness and systemic perfusion more effectively than standard ACLS measures [1,3,5,6].

## Conclusions

This report underscores that BRASH syndrome is not a coincidental coexistence of bradycardia, renal dysfunction, AV-nodal blockade, shock, and hyperkalemia, but rather a self-perpetuating cycle demanding integrated management. Timely recognition with immediate correction of hyperkalemia, withdrawal of culprit agents, renal optimization, and stabilization of perfusion form the cornerstone of therapy. While many patients recover with conservative measures, advanced interventions such as temporary pacing may become necessary; however, pacing is rarely effective until electrolyte and metabolic disturbances are corrected. Preventive efforts, including thorough medication reconciliation and close electrolyte monitoring in high-risk patients, are vital to reduce recurrence. Greater awareness and prospective multicenter studies are warranted to establish standardized diagnostic criteria, refine treatment strategies, and develop evidence-based management guidelines for BRASH syndrome.
